# A European approach to infection prevention and control goals

**DOI:** 10.3205/dgkh000400

**Published:** 2021-11-02

**Authors:** Martin Exner, Bärbel Christiansen, Roberto Cocconi, Alexander Friedrich, Philippe Hartemann, Peter Heeg, Ursel Heudorf, CaroIa llschner, Axel Kramer, Wolfgang Merkens, Peter Oltmanns, Frank Pitten, Hans-Günther Sonntag, Kathrin Steinhauer, Athanassios Tsakris, Rolanda Valinteliene, Violeta Voynova-Georgieva

**Affiliations:** 1 Institute of Hygiene and Public Health, Bonn University Hospital, Bonn, Germany; 2 Department of Internal Hygiene, Schleswig-Holstein University Hospital, Kiel, Germany; 3 Azienda Sanitaria, Universitaria Integrata di Undine, Udine, Italy; 4 Universitair Medisch Centrum Groningen, Groningen, The Netherlands; 5 Departement Environnement et Santé Publique S.E.R.E.S., Faculté de Médecine, Nancy, France; 6 Senior Consultant in Hygiene and Infection Control, Ammerbuch, Germany; 7 Public Health Department, City of Frankfurt, Frankfurt, Germany; 8 Institute of Hygiene and Environmental Medicine, University Medicine Greifswald, Greifswald, Germany; 9 Schülke & Mayr GmbH, Norderstedt, Germany; 10 IKI - Institut für Krankenhaushygiene & Infektionskontrolle GmbH, Gießen, Germany; 11 Institute of Hygiene and Medical Microbiology, University of Heidelberg, Heidelberg, Germany; 12 Department of Microbiology, Medical School, University of Athens, Athens, Greece; 13 Visuomenes sveikatos technologiju centro vadove, Higienos institutas, Vilnius, Lithuania; 14 Department of Epidemiological Surveillance and Analysis, Sofia, Bulgaria

**Keywords:** hygiene, Europe, infection prevention, infection control, pandemic, COVID-19, antibiotic-resistant bacteria, environmental disaster

## Abstract

The current pandemic caused by COVID-19 has underlined the importance of a joint effort and approach to ensure patient and health care worker safety in medical care throughout Europe.

In addition, the recent flood disasters in Germany and other countries called for immediate joint action, in this case with regard to the prevention of water-borne infections. Environmental disasters will increase with consequences for hospitals and nursing homes.

Cooperative efforts are needed for preventing and controlling associated infection outbreaks, new pathogens will appear and a geographic shift of infectious diseases previously not detected in certain areas has already been observed.

This approach to infection prevention and control must entail structural as well as regulatory aspects. The principle of equal protection against infections in all European countries must be implemented. Prevention and control of infections, including nosocomial infections, infections caused by antibiotic-resistant bacteria as well as pandemics, need to be based on equal standards in all of Europe.

Protection against infections and other public health risks in all European countries is the best guarantor for building trust and identification of citizens in our common Europe.

Experts in the fields of hygiene, microbiology, infectiology and epidemiology have to pool the expertise on the prevention and control of infections from different European countries and define key targets for achieving a high standard of hygiene measures throughout Europe. The participants of the Rudolf Schülke Foundation International Symposium call for immediate action and priority to be given to the realization of the proposed 16-point plan.

## Zusammenfassung

Die aktuelle COVID-19-Pandemie hat deutlich gemacht, wie wichtig gemeinsame Anstrengungen und ein gemeinsames Vorgehen sind, um die Sicherheit von Patienten und Mitarbeitenden in der medizinischen Versorgung in ganz Europa zu gewährleisten.

Auch die jüngsten Hochwasserkatastrophen in Deutschland und anderen Ländern haben gezeigt, dass sofortige überregionale Maßnahmen, in diesem Fall zur Prävention von durch Wasser übertragenen Infektionen, dringend erforderlich sind. Umweltkatastrophen werden zunehmen, und sie haben auch für Krankenhäuser und Pflegeheime Folgen.

Ausbrüche müssen verhindert und unter Kontrolle gebracht werden. Zugleich ist mit dem Auftreten neuer Krankheitserreger sowie mit einer bereits jetzt zu beobachtenden geographischen Verschiebung von Infektionskrankheiten zu rechnen.

Der Ansatz zur Prävention und Bekämpfung von Infektionskrankheiten muss sowohl strukturelle als auch politische Aspekte beinhalten. Es gilt, den Grundsatz des gleichen Schutzes vor Infektionen in allen europäischen Ländern umzusetzen. Prävention und Bekämpfung von Infektionen, einschließlich nosokomialer Infektionen, Infektionen durch antibiotikaresistente Bakterien sowie Pandemien, müssen in ganz Europa auf den gleichen Standards beruhen.

Der Schutz vor Infektionen und anderen Risiken für die öffentliche Gesundheit in allen Ländern Europas ist der beste Garant für die Schaffung von Vertrauen und Identifikation der Bürger in unserem gemeinsamen Europa.

Expertinnen und Experten auf den Gebieten der Hygiene, Mikrobiologie, Infektiologie und Epidemiologie müssen ihre Expertise zur Prävention und Bekämpfung von Infektionen aus verschiedenen europäischen Ländern bündeln und die wichtigsten Ziele zur Erreichung eines hohen Standards von Hygienemaßnahmen in Europa definieren. Die Teilnehmer des Internationalen Symposions der Rudolf Schülke Foundation fordern, umgehend zu handeln und der Umsetzung des vorgeschlagenen 16-Punkte-Plans Priorität einzuräumen.

## Memorandum of European hygiene specialists

### This house is on fire: Coronavirus will rise and fall, infectious pathogens will remain

On February 28, 2020, hygiene experts from various European countries were invited by the Rudolf Schülke Foundation, Hamburg, to discuss the current strengths and weaknesses in infection precautions throughout Europe and strategies for improvement. Prof. Dr. med. Martin Exner, chairman of the Foundation, declared his vision: 

**Every European in every European country should have the right to be medically cared for according to the same hygiene safety standards.**

In the meantime, during the course of the COVID-19 pandemic, this vision of equal protection for all has gained even more significance and urgency.

The message is clear: ***This house is on fire*.** This imagery was originally introduced by Professor Alexander Friedrich, Groningen, for describing the state of urgency to take action against the ongoing spread of Gram-negative pathogens throughout Europe in order to illustrate that infection prevention and control must be a cooperative effort across borders. It was introduced some time ago, without corona wildfires. 

However, independent of epidemics like SARS, swine flu, COVID-19, and the threat posed by antibiotic-resistant microorganisms, it is – now more than ever – evident that infections **constitute a major public health threat**. It is a threat which is not confined to certain settings like hospitals or to specific geographical locations. It is a threat which concerns us all. And it is a threat which must be dealt with on a European level in order to ensure that all European citizens can rely on the implementation of minimum standards of infection prevention and control measures.

Although there have been efforts to combat antibiotic resistance and to ensure quality of care, including the prevention and control of healthcare-associated infections by the European Council, the fire has not been prevented from spreading and new fires have started. Thus, **infection prevention and control must become a mandatory health-related target,** requiring constant evaluation, adaptation and revision. Antimicrobial resistance and emergence and re-emergence of new pathogens must be seen as interconnected. Even though interventions need to take into account regional or local needs and preconditions in order to be successful, a European-wide approach is indispensable.

The authors of this statement view the following **infection-related goals and targets as a priority for EU health policies and call for immediate action**:

1. Define specific, concrete obligatory infection prevention goals with concrete time frames (e.g., make Europe CRE-free by 2030).
2. Define standards for binding regulatory frameworks in each member state to ensure compliance with essential recommendations related to infection prevention issues.
3. Define standards for obligatory availability of a sufficient number of tertiary academic institutions (universities) to ensure professional education in hygiene of medical doctors and nurses during their studies, to be able to offer further education for specialists in hygiene, public health, global health and related fields, and to conduct research.
4. Establish a framework for the implementation of best practice for combatting antimicrobial resistance in each European country.
5. Define key hygiene indicators and scores for minimum requirements which must be met for accreditation of inpatient and outpatient medical care settings (including long-term-care facilities), methods for monitoring compliance, and sanctions for non-compliance.
6. Establish standard requirements and directives for professional cleaning and disinfection of medical care facilities.
7. Ensure continuing revision and enforcement of medical device processing regulations.
8. Establish minimum standards for adequate structural and building requirements.
9. Empower ECDC to pay visits to each member state to monitor hygiene processes according to common core strategies.
10. Ensure surveillance infrastructure and reliable evaluation of data in each European country and comparability across countries.
11. Ensure outbreak management according to minimum standards in each European country.
12. Systematically educate the general public on basic hygiene and infection prevention, starting from childhood in each European country.
13. Adopt a one health approach.
14. Promote production of antibiotics, disinfectants and other essential infection control equipment within Europe.
15. Promote research and development of antibiotics and disinfectants within Europe.
16. Use expert knowledge and existing guidelines, publications and curricula to find a consensus and implement an obligatory framework for a European infection control program.

20 October, 2021

## Notes

### Competing interests

The authors declare that they have no competing interests.

### Contact information

For information on the presentations held during the symposium of the Rudolf Schülke Foundation, please contact Andrea Rodewald:

andrea.rodewald@schuelke.com

